# Color Stability of Bulk-Fill Flowable Resin Composites After Artificial Aging

**DOI:** 10.3390/dj12110350

**Published:** 2024-10-31

**Authors:** Franciele Floriani, Carlos A. Jurado, Nikkita Madhu, Mark A. Lackey, Francisco X. Azpiazu-Flores, Guilherme Carpena Lopes

**Affiliations:** 1 Department of Prosthodontics, The University of Iowa College of Dentistry, Iowa City, IA 52241, USA; 2 Department of General Dentistry, The University of Tennessee Health Science Center College of Dentistry, Memphis, TN 38103, USA; 3 Division of Restorative and Prosthetic Dentistry, The Ohio State University College of Dentistry, Columbus, OH 43210, USA; 4 Department of Operative Dentistry, Federal University of Santa Catarina, Florianopolis 88040, SC, Brazil

**Keywords:** resin composite, bulk-fill, color, flowable composite, shade

## Abstract

Background: This study aimed to evaluate the color stability of bulk-fill flowable resin composites with 2 difference shades at baseline and after artificial aging. Methods: Disk-shaped specimens (Ø10 × 4 mm) were fabricated from three bulk-fill flowable resin resin composites (Filtek Bulk-Fill Flow, Venus Bulk-Fill Flow, and Estelite Bulk-Fill Flow). The specimens in each bulk-fill resin composite group were divided into two subgroups (n = 10 per subgroup) with two different shades, A1 (N = 30) and A3 (N = 30), and were polymerized with light curing (800 mW/cm^2^/Valo LED Unit, Ultradent) and polished. The color difference between bulk-fill resin composites was evaluated at baseline and after artificial aging using a spectrophotometer (CM-700d, Konica Minolta, Tokyo, Japan) under D65 illumination. Color coordinates were measured with CIEDE2000, and color differences (∆E_00_) and relative translucency parameter (RTP) values were calculated. Subsequently, the comparison of color changes (∆E_00_) before and after thermocycling was performed using the *t*-test for paired samples. Results: The bulk-fill flow resin composites evaluated in the present study were capable of mimicking important optical properties such as light transmission. All the resin composites provided acceptable color stability at baseline and after thermocycling when the color A1 was used. On the other hand, whenever the shade A3 was used, the Venus Bulk-Fill Flow demonstrated the best optical properties. There was no statistically significant difference when comparing baseline and after thermocycling in bulk-fill flowable resin composites (*p* > 0.05). After thermocycling, A1 bulk-fill flowable resin composites provided acceptable color stability, and all A3 bulk-fill flowable resin composites provided visible color change, except for the Venus Bulk-Fill Flow (∆E_00_ = 2.35). Conclusions: Estelite Bulk-Fill Flow displayed the best color stability (∆E_00_ = 2.22) between all the combinations evaluated.

## 1. Introduction

Recently, a new group of restorative material known as “bulk-fill resin composites” has been developed, offering enhanced depth of cure and minimal polymerization shrinkage [1,2,3]. These advancements were achieved by modifying the filler content, adjusting the filler size relative to the light wavelength, and optimizing the refractive index between the inorganic and organic components. Bulk-fill resin composites can be applied in increments of up to 4.0 mm, providing satisfactory mechanical properties while avoiding the disadvantages associated with the traditional incremental technique [4,5,6,7,8,9,10,11].

Currently, two types of bulk-fill resin composites are commercially available: full-body bulk-fill and flowable bulk-fill resin composites [12]. The full-body bulk-fill resin composites have a high inorganic filler loading, resulting in a highly viscous consistency. For this reason, they are also referred to as paste-like or sculptable bulk-fill resin composites. Their resulting volumetric shrinkage and residual stress showed promising outcomes compared with regular methacrylate-based resin composites. The flexural strength and wear resistance have been compared to those of conventional resin composites, and the creep has been reported as clinically adequate [13]. To provide good marginal adaptation and reduce the polymerization stress, low-viscosity bulk-fill composites were produced. These materials made possible to build up a 4 mm composite layer in one increment, with optimum adaptation, while reducing working time through a simple technique. Nowadays, large cavities in posterior teeth are commonly restored with multiple layers of universal resin composites, conventionally layered in 2 mm increments, or with bulk-fill resin composites, applied in 4 to 5 mm thick layers. However, sculptable bulk-fill composites rely in terms of esthetics, on a single layer as well as on fewer shades than universal composites [13].

Although these low-viscosity bulk-fill composites are very practical restorative materials, their esthetic and mechanical properties are not sufficient to place them on the surface; they must be covered with a layer of medium-viscosity conventional composite resin [12]. Consequently, products in this category (such as Surefil^®^ SDR™ flow, Dentsply Sirona, Charlotte, NC, USA) are preferably used for the direct restoration of deep cavities in posterior teeth [3]. Currently, SDR^®^ flow+ has been developed with improved wear resistance, and with four different shades, allowing it to be placed without an additional capping layer. This broadens its indications to include the restoration of class III and V cavities [4]. Using a low-viscosity bulk-fill composite in the anterior region represents cutting-edge practice. However, composites placed on the surface must meet multiple criteria, such as high resistance to abrasion, wear resistance, hardness, tensile strength, shade stability, gloss, and low surface roughness after finishing and polishing [5].

On the other hand, flowable bulk-fill resin composites generally have a lower inorganic filler content than full-body bulk-fill composites [14]. Their lower viscosity allows for better adaptation to the cavity walls and easier application in small cavities with syringe tips. However, their inferior mechanical properties compared to highly filled nanohybrid composites warrants caution when used in areas subject to high occlusal load [14]. Recent studies have shown lower wear resistance and strength in these materials, thus suggesting that restorations under high occlusal stress should be capped with a high-viscosity conventional composite [14]. Flowable bulk-fill resin composites are commonly used as a base layer, followed by a capping layer of conventional resin composites, with recent clinical evaluations demonstrating acceptable outcomes in Class I and II cavities [15]. Nonetheless, presently concern remain regarding using these composites resins without a capping layer at the restoration surface, and limited research exists regarding their optical characteristics when used alone.

This study aimed to evaluate the color stability of bulk-fill flowable resin composites at baseline and after artificial aging by comparing them with two different materials. The null hypothesis posited that the color stability of different bulk-fill flowable resins would be influenced by artificial aging.

## 2. Materials and Methods

The sample size calculation was performed using previous studies as a reference [16] and resulted in 10 specimens for each bulk-fill flowable resin composite evaluated. A total of 60 disk-shaped specimens (Ø10 × 4 mm) were made using three bulk-fill resin composites (1) Filtek Universal Bulk-Fill Flow (3M Oral Care, St. Paul, MN, USA), (2) Venus Bulk-Fill Flow (Kulzer, Hanau, Germany) and (3) Estelite Bulk-Fill Flow (Tokuyama Dental Corporation, Tokyo, Japan) with two different shades, A1 (N = 30) and A3 (N = 30).

### 2.1. Specimen Preparation

A two-piece Teflon mold of Ø10 mm and 4 mm thickness was used to make the disk-shaped composite specimens A cellophane sheet was placed over the mold and pressed uniformly. Extra flash was removed, until the material was made flush with the top surface of the Teflon mold (R = 3.0 mm). All specimens were prepared using a high-speed handpiece (Synea Vision TK 94, W&H Dentalwerk Bürmoos GmbH, Bürmoos, Salzburgo, Austria) with abundant water irrigation and a spherical diamond bur (bur head ø = 1.5 mm, # 1012, KG Sorensen, Barueri, SP, Brazil). The windows on the removable plates were used as a guide to standardize the cavity preparation with the diamond bur, and dimensions were verified using a digital caliper (Teknikel, Istanbul, Turkey) and a periodontal probe until the following preparation dimensions were achieved: mesio-distal = 3.0 mm, cervical-occlusal = 3.0 mm, and depth = 1.5 mm.

All specimens were polished with polishing disks (Zenit Flex Pop On; President Dental GmbH, Munich, Germany) under dry conditions using a slow-speed handpiece (10,000 rpm). To ensure the accuracy of the results, the specimens were carefully prepared by rinsing and drying between each step to remove any debris from polishing. Additionally, new polishing materials were used for each composite specimen. The resin composite specimens were stored in distilled water in a dark bottle at 37 °C (±1 °C) for 24 h in an incubator until testing

### 2.2. Color Evaluation

**Figure 1:**
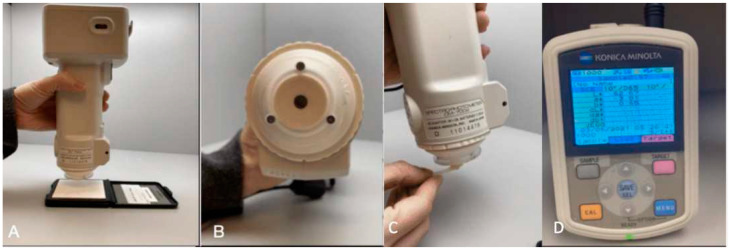
Spectrophotometer CM-700d (Konica Minolta, Japan). (**A**) White balance calibration with the calibration plate provided by the manufacturer before color analysis. (**B**) Diameter of light color specimen’s measurements in all analyses according to the size of the specimen. (**C**) Standardized color evaluation on top of the tooth with Class V restorations. (**D**) The magnitude CIELab color parameters, L*, a*, and b*, for each specific specimen.

The color analysis was performed by a single operator, using a digital spectrophotometer (CM-700d, Konica Minolta, Japan) inside a laboratory light cabin (Macbeth Judge II D65 light; Hong Kong, China) (Figure 1) to produce simulated daylight (D65), (Figure 1A). A 3.0 mm aperture size was used according to specimen diameter (Figure 1B). The color was evaluated in the same position for all specimens (Figure 1C). On the screen, the magnitude CIELab color parameters, L*, a*, and b*, were measured for each specific specimen (Figure 1D). Each measurement was repeated three times, and the average value was recorded.

To calculate CIEDE2000, the color difference in each inherent color parameter was determined as ∆L, ∆a, or ∆b by subtracting each pre-aged and immediately post-aged coordinate parameter value (+a* = red, −a* = green; +b* = yellow, −b* = blue; +L* = white, −L* = black). Considering that the K_L_ parametric factor in CIEDE2000 is recommended to have a value of 1 under reference conditions, K_L_, K_C,_ and K_H_ were the parametric values for lightness, chroma, and hue, which were all set to 1 [17]. The color difference between baseline and after thermocycling (after aging) was calculated using the CIED2000 formula (∆E_00_) using a spectrophotometer [16]:

$${∆E}_{00}={\left[{\left(\frac{∆L}{K_{L}S_{L}}\right)}^{2}+{\left(\frac{∆C}{K_{C}S_{C}}\right)}^{2}+{\left(\frac{∆H}{K_{H}S_{H}}\right)}^{2}+R_{T}\left(\frac{∆C}{K_{C}S_{C}}\right)\left(\frac{∆H}{K_{H}S_{H}}\right)\right]}^{1/2}$$

### 2.3. Measurement of Relative Translucency Parameter

For the relative translucency parameter (RTP_00_), each resin composite specimen was measured using a spectrophotometer (Lovibond RT Series; Tintometer^®^Group, Lovibond House, Amesbury, Wiltshire, UK) on both black (L*: 1.0, a*: 9.5, and b*:17.5) and white (L*: 89.5, a*: 1.4, and b*: 6.7) backgrounds. The RTP_00_ values of the same specimen were measured before and after thermocycling. The RTP_00_ values were calculated using the CIEDE2000 color formula as follows:

$${RTP}_{00}={\left[{\left(\frac{L_{B}^{\prime }-L_{w}^{\prime }}{K_{L}S_{L}}\right)}^{2}+{\left(\frac{C_{B}^{\prime }-C_{w}^{\prime }}{K_{C}S_{C}}\right)}^{2}+{\left(\frac{H_{B}^{\prime }-H_{w}^{\prime }}{K_{H}S_{H}}\right)}^{2}+R_{T}\left(\frac{C_{B}^{\prime }-C_{w}^{\prime }}{K_{C}S_{C}}\right)\left(\frac{H_{B}^{\prime }-H_{w}^{\prime }}{K_{H}S_{H}}\right)\right]}^{1/2}$$

In the formula above, L, C, and H refer to lightness, chroma, and hue, respectively, while the B (black) and W (white) subscripts indicate the color of the backgrounds these values were obtained on.

After the color difference and relative translucency parameter measurements were taken on all specimens, the specimens were subjected to 10,000 cycles of thermocycling between 5 and 55C with a dwell time of 30 s (THE 1100; SD Mechatronik, Feldkirchen-Westerham, Germany) as reported in previous studies. After thermocycling, new color measurements were performed again on all specimens to assess the color stability (Table 1 and Table 2).

### 2.4. Data Analysis

The two factors analyzed were “resin composite” and “aging process”. A comparison of color changes (∆E_00_) before and after thermocycling was performed using the *t*-test for paired samples and effect size (Cohen’s d) using the spreadsheet. The score for effect size was d = 0.2 small; d = 0.5 mean; and d = 0.8 large [18].

## 3. Results

Table 1 and Table 2 describe the ∆E_00_ values between the different bulk-fill flowable resin composite shades A1 and A3 at baseline/after artificial aging. Table 1 shows that at baseline, there was a correlation between the known clinically acceptable ∆E_00_ of below 2.56, indicating that there is an acceptability threshold (AT), called “acceptable color differences” (AT) or “color tolerances”, between two materials [19]. The present in vitro study evaluated the color tolerances and relative translucency between acrylic teeth and the bulk-fill flowable resin composite at baseline and after artificial aging. Thus, all specimens were in the Filtek Bulk-Fill Flow Universal color A1 (∆E_00_ = 2.15), Venus Bulk-Fill color A1 (∆E_00_ = 2.47), and Estelite Bulk-Fill Flow color A1 (∆E_00_ = 2.12), which showed an acceptable color of tolerance.

Table 2 shows that after thermocycling, the color of bulk-fill resin composite restorations changed, affecting the overall color stability in shades A1 and A3. However, some bulk-fill flowable resin composites were still the acceptable color of tolerance (∆E_00_ ≤ 2.56), namely the Filtek Universal Bulk-Fill Flow color A1 (∆E_00_ = 2.45), Venus Bulk-Fill color A1 (∆E_00_ = 2.37) and Estelite Bulk-Fill Flow color A1 (∆E_00_ = 2.22). Table 3 shows that there was no statistically significant difference in the relative translucency parameter when comparing baseline and after thermocycling in colors A1 and A3 (*p* > 0.05).

## 4. Discussion

This study aimed to evaluate the initial and long-term color stability of bulk-fill flowable resin composites after artificial aging. Three bulk-fill flowable resin composites (Filtek Universal Bulk-Fill Flow [3M Oral Care], Venus Bulk-Fill [Heraeus Kulzer], and Estelite Bulk-Fill [Tokuyama Dental Corporation]) with shades A1 (N = 30) and A3 (N = 30) were assessed. The null hypothesis was rejected (*p* < 0.05), as significant differences were observed in the color stability of these materials at baseline and after thermocycling.

Previous studies have considered bulk-fill resin composites as viable options for posterior tooth-colored restorations due to their favorable mechanical and physical properties, which result from their unique chemical composition [17]. For instance, bulk-fill resin composites demonstrated reduced gap formation and improved stress distribution in Class V restorations compared to regular nano-filled composites, regardless of cavity size [9]. Similarly, in Class II restorations, the clinical stability of bulk-fill composites applied in layers up to 4 mm has been comparable to nanohybrid composites after two years [20].

Color stability is a crucial characteristic that reflects the long-term performance and esthetic quality of resin-based materials. Low color stability may indicate a lower degree of polymer conversion, which can contribute to material degradation and compromise the longevity of the restoration. In this study, the A1 shade of bulk-fill flowable resin composites demonstrated clinically imperceptible color changes (ΔE ≤ 2.56) after thermocycling, while shade A3 exhibited visible color changes, except for in Venus Bulk-Fill Flow (∆E_00_ = 2.35), where noticeable clinical differences were observed in.

The translucency parameter of all tested composites decreased after thermocycling, although no statistical significance was found (*p* > 0.05). Although the 4 mm depth in this study did not significantly affect the light curing process, it may have facilitated dye penetration through microleakage, impacting the color stability of the composite [20,21,22].

De Abdulmajeed et al. found that bulk-fill materials exhibited better color stability than conventional composites after thermocycling in specimens with 2 mm thickness [7]. Bulk-fill materials used in posterior areas must withstand high occlusal stresses, and their filler size is positively correlated with properties such as elastic modulus, strength, and hardness [17]. Bulk-fill composites offer a microhardness value between hybrid and flowable composites, but their organic content makes them more susceptible to hydrolytic degradation, water absorption, and reduced color stability [20].

The observed color change can also be attributed to the silane agent present in the resin composite formulation, as its high propensity for water sorption contributes to discoloration. Increasing the filler content improves properties like water absorption, color stability, and wear resistance [20]. Composite shade also plays a role, with darker shades generally showing better color matching due to the presence of pigments, while universal shades may undergo greater color changes due to the absence of pigments [20].

In clinical studies, Balkaya et al. demonstrated acceptable margin discoloration performance in Class II cavities using bulk-fill composites [20]. Oter et al. similarly reported successful clinical outcomes for bulk-fill composites in Class I restorations over one year, particularly in terms of marginal integrity and discoloration [23,24].

Backes et al. compared the color stability of bulk-fill and conventional resin composites after coffee staining, finding that conventional composites exhibited more staining, regardless of the light curing distance [25]. Shamszadeh et al. also reported that universal composites had higher color stability than bulk-fill composites following coffee staining, with discoloration increasing with increment thickness [26].

Considering the potential for patients to have higher thresholds for perceiving color changes than dentists, it may be beneficial to conduct further clinical studies that evaluate the patient’s perspective on color matching and stability. Another important factor is the thickness of the composite layers. While this study used 2 mm specimens, thicker increments may present lower depths of cure, leading to increased staining susceptibility. Future studies should explore the color stability of bulk-fill flowable composites at different thicknesses in clinical settings.

Finally, there are limited data comparing the wear and color stability of bulk-fill flowable resin composites. This study showed that acceptable color stability was more easily achieved in shade A1, likely due to the translucency of the composite material. However, the limitations of this study lie in its in vitro design, and further clinical studies are necessary to evaluate the long-term color stability and translucency of bulk-fill flowable resin composites.

## 5. Conclusions

After thermocycling, A1 bulk-fill flowable resin composites provided acceptable color stability, and all color A3 provided visible color changes, with the exception of Venus Bulk-Fil Flow (∆E_00_ = 2.35). The Estelite Bulk-Fill Flow displayed the best color stability (∆E_00_ = 2.22) between all the combinations evaluated.

## Figures and Tables

**Table 1:** The color difference between bulk-fill flowable resin composite shade A1 and A3 at baseline.

Specimens		Color Difference ∆E_00_ (±SD)
A1	Filtek Bulk-Fill Flow A1	2.15± (0.23) *
A3	Filtek Bulk-Fill Flow A3	3.07± (0.23)
A1	Venus Bulk-Fill Flow A1	2.47± (1.38) *
A3	Venus Bulk-Fill Flow A3	3.45± (1.36) *
A1	Estelite Bulk-Fill Flow A1	2.12± (1.33) *
A3	Estelite Bulk-Fill Flow A3	3.01± (2.33)

* There is a statistically significant difference in comparison to a ΔE_00_ = 2.56.

**Table 2:** The color difference between bulk-fill flowable resin composite shade A1 and A3 after thermocycling.

Specimens		Color Difference ∆E_00_ (±SD)
A1	Filtek Bulk-Fill Flow A1	2.45± (0.23) *
A3	Filtek Bulk-Fill Flow A3	3.37± (1.33) *
A1	Venus Bulk-Fill Flow A1	2.37± (1.25) *
A3	Venus Bulk-Fill Flow A3	2.35± (1.25) *
A1	Estelite Bulk-Fill Flow A1	2.22± (1.23) *
A3	Estelite Bulk-Fill Flow A3	3.11± (2.45) *

* There is a statistically significant difference in comparison to a ΔE_00_ = 2.56.

**Table 3:** Relative translucency parameter between bulk-fill flowable resin composite color A1/A3 before and after thermocycling (TC).

		Baseline		TC		*p*-Value	Cohen’s dz
Material	Color	Mean	±sd	Mean	±sd		
Filtek Bulk-Fill Flow	A1	20.1	0.23	19.5	0.23	0.251	0.92
Venus Bulk-Fill Flow	A1	19.4	1.38	18.7	1.25	0.934	0.05
Estelite Bulk-Fill Flow	A1	18.1	1.33	17.2	1.23	0.933	0.06
Filtek Bulk-Fill Flow	A3	16.0	0.23	15.7	1.33	0.737	0.22
Venus Bulk-Fill Flow	A3	20.4	1.36	19.5	1.25	0.411	0.60
Estelite Bulk-Fill Flow	A3	21.0	2.33	18.1	2.45	0.964	0.03

## Data Availability

The original contributions presented in the study are included in the article, further inquiries can be directed to the corresponding author.
